# Spatiotemporal Distribution of Zika Virus and Its Spatially Heterogeneous Relationship with the Environment

**DOI:** 10.3390/ijerph18010290

**Published:** 2021-01-02

**Authors:** Jie Li, Kun Jia, Yanxu Liu, Bo Yuan, Mu Xia, Wenwu Zhao

**Affiliations:** 1 State Key Laboratory of Remote Sensing Science, Faculty of Geographical Science, Beijing Normal University, Beijing 100875, China; 202021051081@mail.bnu.edu.cn (J.L.); 202031051033@mail.bnu.edu.cn (B.Y.); xiamu@mail.bnu.edu.cn (M.X.); 2 State Key Laboratory of Earth Surface Processes and Resource Ecology, Faculty of Geographical Science, Beijing Normal University, Beijing 100875, China; yanxuliu@bnu.edu.cn (Y.L.); zhaoww@bnu.edu.cn (W.Z.); 3 Institute of Land Surface System and Sustainable Development, Faculty of Geographical Science, Beijing Normal University, Beijing 100875, China

**Keywords:** ZIKV, spatiotemporal distribution, socio-ecological environmental factors, ESDA-GIS model, GWR model, SARS-CoV-2

## Abstract

Infectious diseases have caused some of the most feared plagues and greatly harmed human health. However, despite the qualitative understanding that the occurrence and diffusion of infectious disease is related to the environment, the quantitative relations are unknown for many diseases. Zika virus (ZIKV) is a mosquito-borne virus that poses a fatal threat and has spread explosively throughout the world, impacting human health. From a geographical perspective, this study aims to understand the global hotspots of ZIKV as well as the spatially heterogeneous relationship between ZIKV and environmental factors using exploratory special data analysis (ESDA) model. A geographically weighted regression (GWR) model was used to analyze the influence of the dominant environmental factors on the spread of ZIKV at the continental scale. The results indicated that ZIKV transmission had obvious regional and seasonal heterogeneity. Population density, GDP per capita, and landscape fragmentation were the dominant environmental factors affecting the spread of ZIKV, which indicates that social factors had a greater influence than natural factors on the spread of it. As SARS-CoV-2 is spreading globally, this study can provide methodological reference for fighting against the pandemic.

## 1. Introduction

Global environmental changes have led to the emergence of new infectious diseases at an unprecedented rate [1,2]. Evidence shows that 76 percent of infectious disease pathogens or vectors are affected by climate, and 40 percent of infectious diseases spread faster under global warming [3]. However, despite the qualitative understanding that the occurrence and diffusion of infectious disease relates to the environment, the quantitative relations are unknown for many diseases [4]. ZIKV is an emerging arthropod-borne virus (arbovirus) belonging to the family Flaviviridae and genus Flavivirus [5]. As a zoonotic and highly contagious arbovirus, ZIKV was first detected in 1947 in rhesus monkeys in the Zika jungle of Uganda, and infections had since been reported in Africa, the Americas, Asia and equatorial Pacific Island countries [6]. In 2016, ZIKV spread to 84 countries and regions, and the large-scale outbreak caused substantial losses to families and society, affecting achievements in the work being done to meet the sustainable development goals (SDGs) of the United Nations, especially SDG 3.3 (“By 2030, end the epidemics of AIDS, tuberculosis, malaria and neglected tropical diseases and combat hepatitis, water-borne diseases and other communicable diseases” [7]).

ZIKV is transmitted primarily by the bite of infected mosquito species which are mainly *Aedes aegypti* and *Aedes albopictus* [8]. *A. aegypti* mosquitoes are widely distributed in the tropical and sub-tropical regions, whereas *A. albopictus* mosquitoes are widely distributed throughout the tropical, sub-tropical and temperate regions [9]. Studies have shown that the spread of ZIKV is related to environmental changes, especially climate change [10]. Climate change affects the dynamics of virus transmission by affecting *A. aegypti* and *A. albopictus* and thus changing the epidemiology and models of ZIKV [11,12]. For example, changes in temperature and precipitation were found to alter the spatial distribution and activity intensity of *A. aegypti* and *A. albopictus*, thus altering the incidence of ZIKV [13,14,15,16]. In addition, climate is also an important factor in the geographical spread of infectious diseases, especially zoonoses, and will lead to an increase in ZIKV infections in the currently endemic areas of this virus as well as in other uninfected areas [17]. Besides, environmental changes caused by human activities may lead to the emergence of new infectious diseases [18]. Landscape fragmentation, deforestation and land use change caused by human intervention led to the destruction of mosquitoes’ habitat, and thus they will have frequent activities in human living areas, which results in close contact between wildlife and humans, increasing the risk of the spread of the virus [19,20]. Globalization is also closely related to the spread of ZIKV [21]. Factors such as international migration and economic globalization may also contribute to the global distribution of *A. aegypti* and *A. albopictus*. In this process, the transmission of ZIKV is sophisticated and complex [22]. Therefore, the socio-ecological impacts of environmental factors on the spread of ZIKV cannot be ignored in efforts to prevent and control ZIKV epidemics.

However, because of the geographical heterogeneity of the driving environmental forces, there are few universal quantitative conclusions on the mechanisms by which environmental changes influence ZIKV transmission. Currently, mathematical modeling methods are often used to estimate the transmission risk of ZIKV, in which the effective regeneration number R0 of virus is calculated by developing climate-driven infectious disease model [23,24]. The influence of social economy and climate environment on ZIKV transmission is analyzed by statistical analysis method [25]. Whether the dynamical model of infectious or statistical analysis of data is only from the perspective of the data itself, and lack of spatial cognition in exploring the relationship between environmental factors and ZIKV transmission. From the perspective of geography, considering the heterogeneity of the spatial distribution of environment, this study analyzes the relationship between ZIKV transmission and environmental factors under different conditions, and quantitatively analyzes the influence of environmental factors. Therefore, we intend to identify the global hotspots of ZIKV distribution as well as the spatially heterogeneous relationship between ZIKV and environmental factors with socio-ecological characteristics. Firstly, the spatiotemporal heterogeneity of ZIKV was explored using ESDA-GIS method and seasonal index based on the ZIKV distribution data. Then, the potential environmental factors related to the spread of ZIKV was selected and the dominant environmental factors influencing ZIKV transmission were determined using the ordinary least squares (OLS) method. Finally, a GWR model was built to characterize the relationship between ZIKV transmission and environment factors in a representative continent (South America). As the SARS-CoV-2 pandemic continues to spread, this study can provide experiences for exploring the relationship between SARS-CoV-2 transmission and environmental factors to accelerate the fight against SARS-CoV-2 and achieve the goal of sustainable development sooner.

## 2. Materials and Methods

### 2.1. Study Area

**Figure 1:**
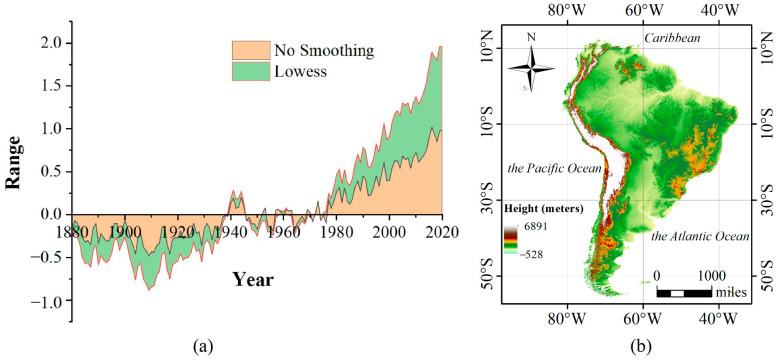
(**a**) Global temperature anomalies from 1880 to 2019. The temperature anomaly is the difference between the average annual temperature since 1980 and the average from 1951–1980. The orange represents data without smoothing, and the green represents data smoothed using the LOWESS (locally weighted scatterplot smoothing) method (**b**) Geographical region of South America. The background is an SRTM digital elevation model (DEM) with a spatial resolution of 250 m.

The study area is divided into two parts: the entire world, over which the spatiotemporal distribution characteristics of ZIKV were analyzed, and South America, where the mechanisms driving the influence of environmental factors on the spread of ZIKV were explored. South America was selected as the study area based on the severity of the outbreak on a global scale, which is detailed in the Results section. In 2017, the global economic growth rate reached 3%, representing a 2.4% increase from 2016. However, global socio-economic development is accompanied by climate change, which has led to continuously warming temperatures from 1880 to 2019, with the highest temperature anomaly (1.02) occurring in 2016 (Figure 1a).

South America is composed of 13 countries with longitudes ranging from 34°46′ W to 81°20′ W and latitudes ranging from 53°54′ S to 12°28′ N (Figure 1b). The landform of South America is heterogeneous, with elevations varying from −528 m to 6891 m. The population density is 21.4 people per square kilometer (statistics for 2011). The dominant climate types are tropical rainforest and tropical savanna. The climate conditions are warm and moist, and the annual average precipitation is approximately 1000 mm.

### 2.2. Data Acquisition and Processing

#### 2.2.1. ZIKV Data

In this study, ZIKV data for major countries from 2015 to 2018 were collected from the websites of the World Health Organization (WHO, Geneva, Switzerland) (https://www.who.int/emergencies/diseases/zika/en/) and the Brazilian National Department of Health Statistics (https://www.gov.br/saude/pt-br). Statistical and spatial analyses were conducted based on the global distribution of ZIKV. As ZIKV data for Africa were not provided by the WHO and relevant statistical agencies, this continent was not considered.

**Figure 2:**
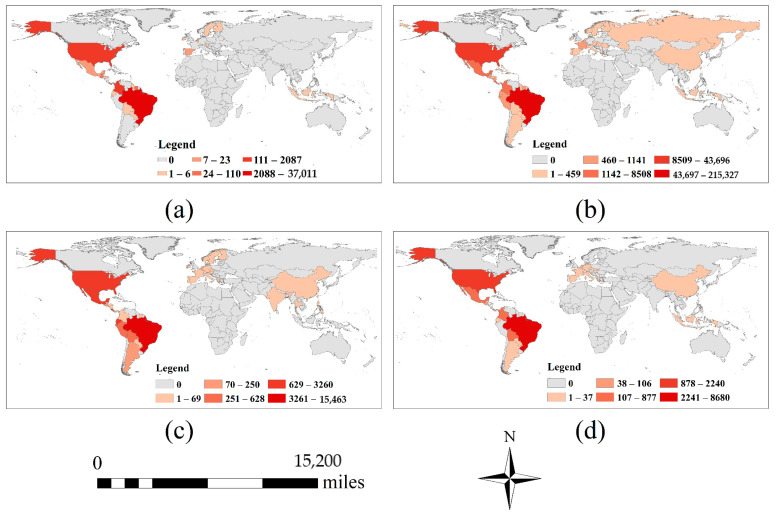
The spatial distribution of ZIKV from 2015 to 2018. The sub-graph (**a**–**d**) represent 2015, 2016, 2017 and 2018 respectively. The base map does not include Antarctica, as no cases have been reported there.

The global ZIKV spatial distribution (Figure 2) shows that the confirmed ZIKV cases are mainly concentrated in South America, where ZIKV is widespread in almost every country. Therefore, it is crucial to study the relationship between the spread of ZIKV and corresponding environmental factors in South America, the area most affected by ZIKV. The time of the outbreak (2016) was selected as the study period, which is detailed in the Results section.

#### 2.2.2. Environmental Data

Weather and climate change influence mosquito geographic distribution, population abundance, lifespan, and transmission potential [25]. The research shows that the temperature range of ZIKV transmission is 18~34°, and it peaks at 29° [26]. In the context of global warming, the annual temperature is likely to rise to the optimal value for ZIKV transmission. In 2015, Brazil’s winter and spring were the warmest and driest on record, with higher winter temperatures contributing to ZIKV’s propagation and spread [10]. Studies have shown that ZIKV-linked microcephaly cases has an obvious correlation with the percentage of forest coverage [27]. The high density of forest cover was negatively correlated with the prevalence rate of ZIKV, which could be interpreted as deforestation and destruction of habitat led to a deterioration of landscape fragmentation. Meanwhile, population density changes from a lower level to a higher level, expanding the risk of ZIKV transmission. In addition, in terms of socio-ecological factors, studies have pointed out that higher GDP and health conditions reduce the transmission of ZIKV [28].

**Figure 3:**
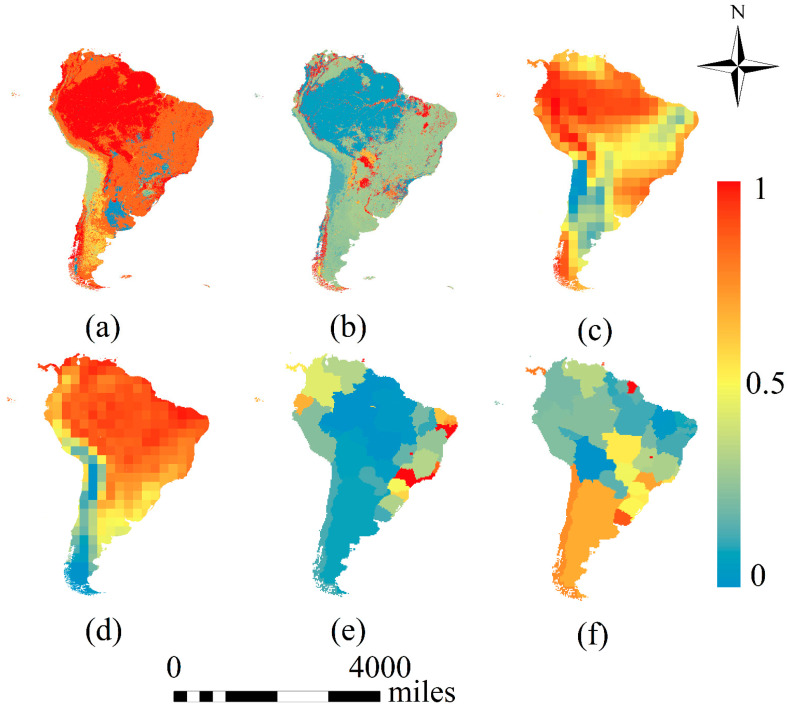
Normalized environmental factors affecting the spread of ZIKV. Population density and GDP per capita are measured by country (by state in Brazil), while other data are measured at the pixel scale. (**a**–**f**) represent biodiversity, landscape fragmentation, relative humidity, temperature, population density and GDP per capita respectively.

Based on above, meteorological, social and ecological factors were all taken into account as environmental factors in this study, the variables of which included temperature, relative humidity, population density, GDP per capita, biodiversity and landscape fragmentation (Figure 3). The meteorological data consisted of the monthly dataset from the National Centers for Environmental Prediction (NCEP) 2016 reanalysis data and were obtained from the National Oceanic and Atmospheric Administration (NOAA) (https://psl.noaa.gov/data/). The 2016 land use data used are available in the Land Processes Distributed Active Archive Centre (LP DAAC, located at the U.S. Department of the Interior, U.S. Geological Survey (USGS) Earth Resources Observation and Science (EROS) Center in Sioux Falls, SD.) products (https://lpdaac.usgs.gov/products/mcd12q1v006/#tools), and the 2016 social statistics were mainly collected from the World Bank (https://databank.shihang.org/home.aspx) and Brazilian National Geographic and Statistics Agency (https://www.ibge.gov.br/).

The value of habitat quality is used to describe biodiversity. A higher habitat quality value indicates richer biodiversity, and richer biodiversity indicates higher habitat quality [29]. In this study, the Integrated Valuation of Ecosystem Services and Trade-offs (InVEST 3.8) model was used to calculate habitat quality by analyzing land cover and the degree of threat to biodiversity [30]. The formula for calculating habitat quality is as follows:

(1) $$Q_{xj}=H_{j}\left[1-\left(\frac{D_{xj}{}^{z}}{D_{xj}{}^{z}+k^{z}}\right)\right]$$

where *Q_xj_* is the habitat quality index of landscape type *j* in grid unit *x*, *H_j_* is the habitat suitability score of landscape type *j*, *z* and *k* are constants, and *D_xj_* is the habitat degradation index, indicating the degree of degradation of a habitat after being stressed.

The degree of fragmentation of the landscape is expressed by patch density. A larger patch density indicates smaller patches and a higher degree of fragmentation. In this study, FRAGSTATS 4.2 software (the authors at the University of Massachusetts, Amherst, MA 01003, USA), used for calculating ecological parameters, was applied to measure the degree of landscape fragmentation by analyzing land cover types. The formula for calculating patch density is as follows [31]:

(2) $$PD=\frac{N}{A}*10000*100$$

where *N* is the total area of the *i*th type of landscape element, *A* is the total area of the landscape, *PD* > 0, and the unit is patches per square kilometers.

### 2.3. Methods

**Figure 4:**
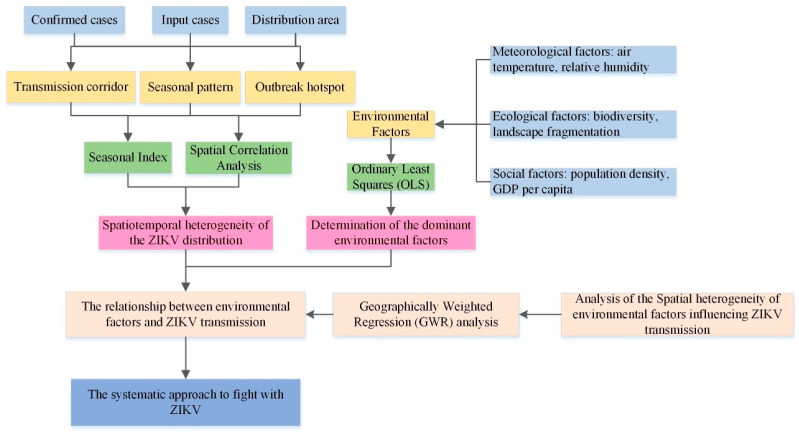
The research flowchart.

To explore the mechanisms by which environmental factors influence ZIKV transmission, the spatiotemporal distribution characteristics of ZIKV transmission were analyzed by using ESDA-GIS method which includes spatial correlation analysis and hotspot analysis, as well as seasonal index method, then the dominant environmental factors contributing to that transmission were determined, and finally, a quantitative model of the relationship was constructed (Figure 4). In this study, the variance inflation factor (VIF), based on OLS method, was used to determine the dominant environmental factors from the initially selected factors of temperature, relative humidity, landscape fragmentation, biodiversity, population density, and GDP per capita. Based on the environmental factors determined to be dominant, a GWR model was used to explore the relationship between the environment and ZIKV transmission, and the geographical differences in the effects of the environment on ZIKV transmission in different regions were also analyzed. This study highlights a direction for studying the relationship between ZIKV epidemic and the environment social-ecological environment factors in geography-based method.

#### 2.3.1. Spatiotemporal Analysis of ZIKV Epidemic

Exploratory spatial data analysis (ESDA) is used to analyze the spatiotemporal distribution of ZIKV epidemic. Based on GIS technology, spatial analysis of geographic information is combined with the spread of infectious diseases to explore the spatial distribution pattern of ZIKV. In this study, the ESDA-GIS analysis model was used to explore the spatial clustering and differentiation of ZIKV transmission from the perspectives of hotspot analysis and spatial correlation analysis, and reveal the rule of its spatiotemporal distribution. 

Hotspot analysis (Getis-Ord Gi*) can express the features of cold spots and hot spots in a certain regional spatial clustering, and intuitively display the hot spots and cold spots within the scope of the research area. It identifies statistically the clusters of points having higher magnitude using the quantity of Relative Risk Ratio [32]. Moran’s Index (Moran’s I) statistics was used to evaluate autocorrelation in ZIKV spatial distribution and test how regions were clustered or dispersed in space [33]. The LISA cluster index is the indicator of spatial association. The localities that show statistically significant (0.05 level) cluster of high values are indicated by (HH) and the localities that show statistically significant (0.05 level) cluster of low values are indicated by (LL). A low negative z score (for example, *z* < −1.96) for a locality indicates if the locality has a high value and is surrounded by locality with low values (HL) or if the locality has a low value and is surrounded by localities with high values (LH) [32].

Seasonal index is a method to describe the seasonal change of time series based on the characteristic of seasonal cycle variation. The seasonal index method analyzes and forecasts the data according to the seasonal regularity. The SPSS v26.0 (IBM, Armonk, NY, USA) software is used to calculate the seasonal index of ZIKV from 2015 to 2018.

#### 2.3.2. Determination of the Dominant Environmental Factors

The OLS model [34] is a global regression method and was selected to determine the dominant environmental factors influencing ZIKV transmission. As a common mathematical optimization method, OLS makes estimations by minimizing the residual sum of squares. By performing OLS linear regression, the relationship between a dependent variable and a set of explanatory variables can be modelled. The regression model is expressed as:

(3) $$Y_{i}=\hat{\beta_{0}}+\hat{\beta_{1}}X_{i}+e_{i}$$

where *Y_i_* represents the observed value of dependent variable *Y* of individual *i*, *X_i_* is the observed value of explanatory variable *X* of individual *i*, *β*_0_ is the intercept, *β*_1_ is the coefficient of *X_i_*, and *e_i_* is the sample error (*X_i_*, *Y_i_*). In this study, the explanatory variables are the environmental factors, and the dependent variable is the number of confirmed ZIKV cases. The VIF (based on the OLS regression) was used to test the linear correlation between these factors. The principle of this method is to use N other factors for the explanation of the regression of each factor. The formula is as follows:

(4) $$VIF_{i}=\frac{1}{1-R_{i}^{2}}$$

where *R_i_* is the complex correlation coefficient between the *i*th variable (*X_i_*) and all the other variables (*X_j_*) (*i* = 1, 2, …, *k*; *i* ≠ *j*). A small *R_i_* indicates a low linear correlation between factors.

#### 2.3.3. Geographical Weighted Regression Model

The GWR model [35] was selected to explore the quantitative relationship between ZIKV transmission and the dominant environmental factors. GWR, as an extension of the traditional least squares’ regression model, is a partial regression method. According to the first law of geography, the closer the distance is between locations, the greater the correlation between them. Therefore, for the confirmed ZIKV cases and related environmental factors in a given geographical location, a GWR model, which accounts for geographical differentiation, can be used to build the relationship between the dependent variable and the explanatory variables. GWR models use spatially weighted matrices to assign weights to explanatory variables at each spatial location and estimate the relationship between the variables based on their spatial variability. The regression model is expressed as:

(5) $$Y_{i}=\beta_{0}\left(u_{i},v_{i}\right)+\overset{p}{\underset{j=1}{\sum }}\beta_{j}\left(u_{i},v_{i}\right)x_{ij}+\varepsilon$$

where (*Y_i_*; *x_i_*_1_, *x_i_*_2_…, *x_ip_*) are the observed values of the dependent variable (*Y*) and the independent variables (*x*_1_, *x*_2_…, *x_p_*) at a geographic location (*u_i_*, *v_i_*) (*i* = 1, 2…, *n*). *β_j_* (*u_i_*, *v_i_*) (*j* = 0, 1…, *p*) represents the unknown parameters at the observation point (*u_i_*, *v_i_*), and *ε_i_* (*i* = 1, 2…, *n*) is an independent and identically distributed random error, which is usually assumed to obey N (0, σ^2^).

## 3. Results

### 3.1. Characteristics of the Spatiotemporal Distribution of ZIKV

**Figure 5:**
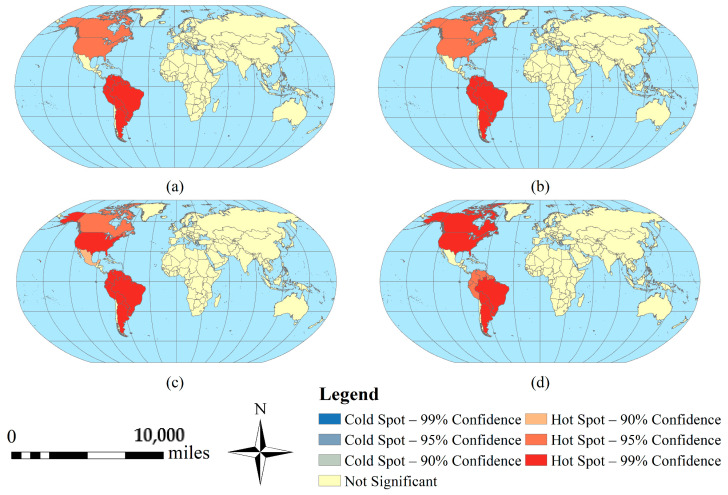
Hot spots of ZIKV epidemic. The sub-graph (**a**–**d**) represent 2015, 2016, 2017 and 2018 respectively.

**Figure 6:**
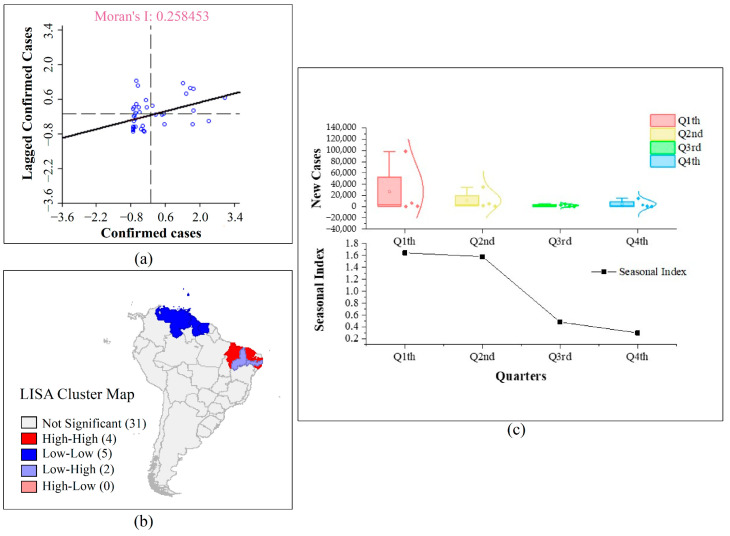
Spatial correlation in South America. The sub-graph (**a**–**c**) represent Moran index, LISA Cluster Map and seasonal index of four quarters respectively.

According to the spatiotemporal distribution of ZIKV (Figure 2), hotspot analysis was used to identify the hot spots of ZIKV epidemic. Figure 5 shows that South America has been severely affected by ZIKV from 2015 to 2018. Meanwhile, due to its geographically proximity, North America was second in the extent of the damage, and other continents and regions of the world were less affected by ZIKV. Using Moran’s I to analyze the spatial correlation of the confirmed ZIKV cases in South America, it can be found that the value of Moran’s I is about 0.26, indicating that the confirmed ZIKV cases have an obvious positive correlation in the geographical location (Figure 6a). It means that the epidemic has a spatial clustering feature. It is more clearly shown in LISA Cluster Map (Figure 6b) that there is a high (HH) clustering in Alagoas, Ceara, Maranhao and Paraiba in Brazil, account for 9.5% approximately, low and high (LH) clustering in Pernambuco and Piaui in Brazil, account for 4.8% approximately, and low (LL) clustering in Guadeloupe, Guyana, Suriname, Trinidad and Tobago and Venezuela, account for 11.9% approximately.

**Figure 7:**
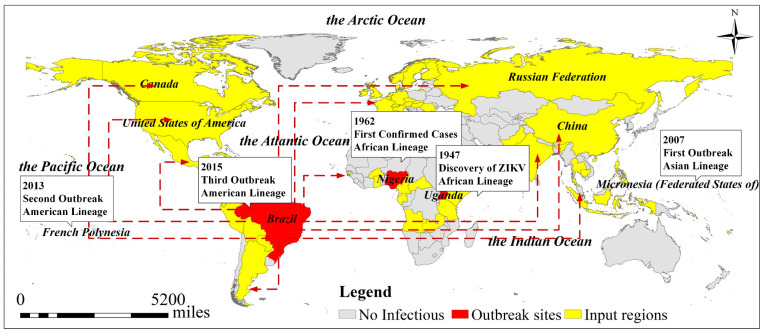
ZIKV outbreak sites and its transmission route in 2016. Yellow represents the input regions, and red represents the outbreak sites. The arrows indicate the ZIKV transmission routes. Information in the box is the outbreak time, outbreak times of ZIKV and virus type [38].

The analysis of the spatial distribution of ZIKV outbreaks over time (Figure 7) shows that the ZIKV outbreak mainly occurred in the tropics around the equator and that the epidemic rapidly spread to many countries and regions around the world, including North America, Europe, Asia and Southeast Asia in 2016, which indicates that the transmission of ZIKV originates from and not limits to *A. aegypti* and *A. albopictus*. With the increasing progress of globalization and global warming, ZIKV is spreading more and more widely around the world. As a highly contagious arbovirus, ZIKV is highly contagious, which can be further spread, particularly via viremic travelers or the movement of infected mosquitoes [36]. Moreover, autochthonous ZIKV transmission as well as cases of ZIKV transmission via physical activity were also been reported [37].

Figure 6c shows the seasonal feature of the confirmed ZIKV cases in South America from 2015 to 2018. The number of new cases in South America differed among the four seasons (Figure 6c). From the result, new cases in the first quarter differed from the other seasons and new cases in the third quarter also differed from the fourth quarter. This showed that the first quarter has the highest new cases among all four seasons in South America. From the seasonal index, the value of the fourth quarters is 1.64%, 1.58%, 0.48% and 0.3% approximately. This could be inferred that the first quarter created favorable environmental conditions for the reproduction and survival of *A. aegypti* and *A. albopictus*, and ZIKV benefits from it. With the enhancement of population mobility, ZIKV cases eventually augmented.

### 3.2. The Geographical Relationship between ZIKV and Environmental Factors

Pearson correlation coefficient was used to analyze the correlation between environmental factors and confirmed ZIKV cases (Table 1). The result indicated that GDP per capita, biodiversity and relative humidity had a negative affect whereas temperature, population density and landscape fragmentation had a positive effect.

Using OLS regression, the VIF values of GDP per capita, temperature, biodiversity, landscape fragmentation, relative humidity and population density were 1.68, 10.61, 55.45, 1.91, 40.33 and 2.54, respectively. The results of the VIF test showed that the explanatory variables of GDP per capita, landscape fragmentation and population density had VIF values less than 7.5; therefore, these variables were selected for the GWR analysis.

Taking the number of confirmed ZIKV cases in South America as the dependent variable and the three dominant environmental factors as independent variables, a GWR model was used to perform the regression analysis. The fitted R2 of the regression model was 0.65, which indicated that the three selected environmental factors are suitable for explaining the variance in ZIKV transmission in South America and that the dominant environmental factors can reliably explain the spatial distribution of the cases.

**Figure 8:**
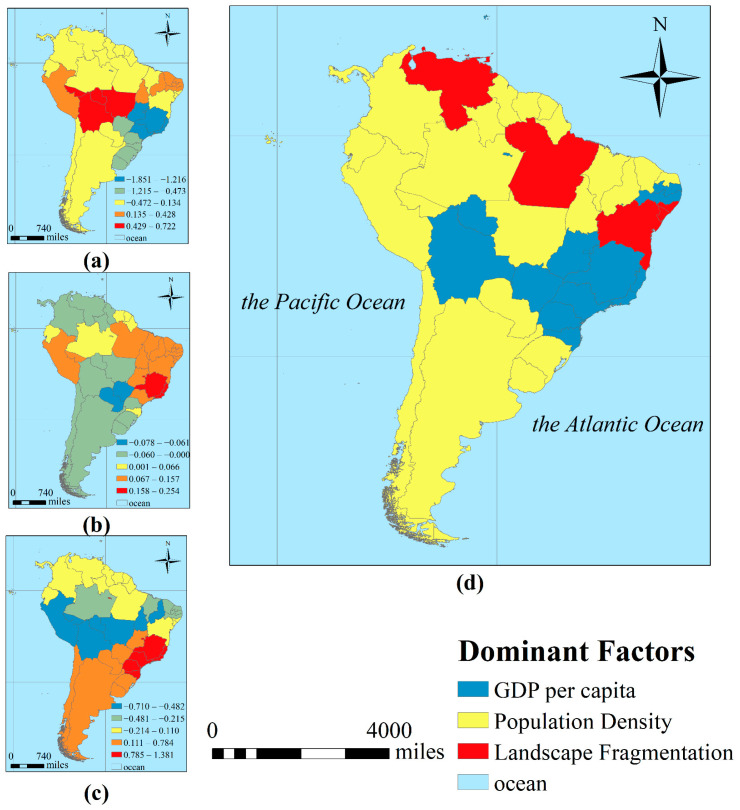
(**a**–**c**) is the regression coefficients of GDP per capita, landscape fragmentation and population density, respectively. (**d**) is the dominant factor impacting different regions.

The average regression coefficients of GDP per capita, population density and landscape fragmentation in the GWR were −0.2, 0.14 and 0.06, respectively, showing that GDP per capita had a negative impact on the number of confirmed ZIKV cases, while population density and landscape fragmentation had positive effects (Figure 8a–c). The results indicated that the higher the GDP per capita is, the higher the level of medical care for the society and the higher the quality of life of the people, which leads to a lower risk of ZIKV infection. Increased population density, which could lead to flexible mobility and closer person-to-person contact, eventually increased the risk of ZIKV transmission. Increasing landscape fragmentation caused by human activities is related to a higher risk of biological habitat destruction and exposure to human activities, which may promote the interaction between wild animals (in this case mainly *A. aegypti* and *A. albopictus*) and humans.

The results show that population density has the widest impact and is the key factor in the spread of ZIKV in South America. The reason may be that high population density increases the risk of people being bitten by infected mosquitoes, and thus spreading the virus worldwide. Domestic and international travel may aggravate the spread of the virus in this region (Figure 8d). GDP per capita is the dominant factor influencing the spread of ZIKV in the states of Bolivia and Brazil, indicating that differences in the socio-economic development of different regions led to the inadequacy of medical supplies in poor and underdeveloped areas and that unbalanced health conditions may accelerate the spread of the virus. The impact of landscape fragmentation on the distribution of ZIKV is manifested in Venezuela, where the ecosystem has been severely damaged in recent years, and in the northeastern and southwestern regions of Brazil.

## 4. Discussion

Based on the data collected for ZIKV and environmental factors, this study analyzed the characteristics of the spatiotemporal distribution and possible transmission routes of ZIKV and quantitatively assessed the impact of environmental factors on ZIKV from a geographical perspective. The analyses revealed that because of the spatial characteristics of ZIKV transmission, different regions may have different prevention and control priorities, and it provides basic rules and data to support the control of ZIKV epidemics. By using quantitative and qualitative analyses, this study has theoretical and practical significance; however, it also has limitations. There is incomplete ZIKV data in some areas, such as Africa, where statistics are unavailable due to the low number of cases; however, this had little influence on this study. Furthermore, some environmental factors which are specific to improve the analysis, such as the population mobility index, lifestyle and religions were not considered; nevertheless, the factors being modelled in the present study allowed a better analysis. These limitations could potentially be overcome in future studies by using open-source data.

As clinical trials of ZIKV vaccines are still underway, more efforts are needed to develop antivirals for ZIKV, and reduce the likelihood of another ZIKV outbreak [39]. Based on the concept of “classification-coordination-collaboration” [40,41], the systematic approach towards fighting ZIKV can be proposed. Authorities should increase vigilance and surveillance toward imported cases of ZIKV infection which would most possibly reduce autochthonous ZIKV transmission and global spread according to the transmission route in 2016 (Figure 7). Moreover, the personal education should be greatly emphasized to make them aware of the potential ways of ZIKV transmission and preventative measures. Based on the dominant factors impacting different regions (Figure 8d), corresponding measures should be taken based on environment-link level. For people in population density-caused areas, they should take precautions against mosquitoes, such as wearing light-colored long-sleeved shirts and trousers, and using insect repellent to exposed parts of their body to minimize vector contact [42]. In areas with ecological environment destructed most, people should pay attention to ecological protection, and relevant departments should carry out ecological management projects to restore the ecology. Besides, public health authorities in ZIKV endemic regions should provide standard healthcare precautions to solve the ZIKV transmission caused by the difference in GDP per capita level.

The analysis of ZIKV shows, patterns of SARS-CoV-2 propagation can be developed and analyzed from a geographic perspective, considering that the heterogeneity of geographical and environmental conditions may cause differences in the transmission of SARS-CoV-2. In addition, by selecting a comprehensive set of environmental factors that influence the spread of SARS-CoV-2, such as the landscape fragmentation, biodiversity index and GDP index selected in this study, the source of the virus can be determined at various levels and the mechanisms driving the influence of environmental factors on the spread of SARS-CoV-2 can be identified.

## 5. Conclusions

In this study, to identify patterns of ZIKV outbreaks and prevent large-scale recurrences, the spatiotemporal distribution characteristics of ZIKV and its spatially heterogeneous relationship with the environment were investigated. The following conclusions can be drawn: (1) ZIKV outbreaks have been mainly concentrated in tropical areas near the equator, and the 2016 outbreak in Brazil was the most serious and quickly spread globally. South America is the area most affected by outbreaks, with high spatial clustering and obvious seasonal patterns. (2) The analysis of the relationship between ZIKV and environmental factors indicated that population density, GDP per capita and landscape fragmentation may be the key factors affecting the dynamics of ZIKV transmission by affecting quantity and distribution of *A. aegypti* and *A. albopictus*. This finding indicated that social factors have a greater influence than natural factors on the spread of infectious diseases. (3) The GWR model can be used to quantitatively analyze the spatial relationships between the environment and ZIKV from a geographic perspective. Population density has the greatest impact on ZIKV occurrence in South America, the influence of GDP per capita is mainly concentrated in a continuous mid-latitude region, and landscape fragmentation has scattered effects in the Amazon Plain and other flat areas. These findings provide a comprehensive description of the ZIKV epidemic in South America and may help to guide subsequent measures to prevent ZIKV transmission.

Furthermore, based on ZIKV transmission patterns and future climate change scenarios, the global risk of ZIKV transmission can be predicted in future studies. Additionally, this study shows that the environment has a non-negligible impact on the spread of ZIKV to a certain extent. Therefore, the conclusions of this study can also provide methodological references for studying the relationship between the SARS-CoV-2 pandemic and the environment. In other words, compared with changes in the natural environment, the impact of human activities plays a vital role in the emergence and development of infectious diseases.

## Figures and Tables

**Table 1:** The correlation coefficients of different environmental factors.

Indicators	GDP Per Capita	Population Density	Temperature	Relative Humidity	Biodiversity	Landscape Fragmentation
Pearson correlation coefficient	−0.047	0.266	0.035	−0.058	−0.133	0.105

## Data Availability

The data presented in this study are available on request from the corresponding author.
